# An immune-related eleven-RNA signature-drived risk score model for prognosis of osteosarcoma metastasis

**DOI:** 10.1038/s41598-024-54292-6

**Published:** 2024-06-11

**Authors:** Jia-Song Teng, Yang Wang

**Affiliations:** 1 The Second Affiliated Hospital of Nanjing University of Chinese Medicine, No. 23 Nanhu Road, Jianye District, Nanjing, 210017 Jiangsu China; 2 Department of Orthopedics, China-Japan Union Hospital of Jilin University, No. 126 Xiantai Street, Changchun, 130033 Jilin China

**Keywords:** Osteosarcoma, Metastasis, Risk score model, Prognosis, Bone cancer, Oncology

## Abstract

This study aimed to determine an immune-related RNA signature as a prognostic marker, in this study, we developed a risk score model for predicting the prognosis of osteosarcoma metastasis. We first downloaded the clinical information and expression data of osteosarcoma samples from the UCSC Xena and GEO databases, of which the former was the training set and the latter was the validation set. Immune infiltration was assessed using the ssGSEA and ESTIMATE algorithms, and the osteosarcoma samples were divided into the Immunity_L and Immunity_H groups. Then, eleven RNAs were identified as the optimal prognostic RNA signatures using LASSO Cox regression analysis for establishing a risk score (RS) model. Kaplan–Meier approach indicated the high-risk group exhibited a shorter survival. Furthermore, we analyzed the tumor metastasis, age, and RS model status were determined to be independent clinical prognostic factors using Cox regression analysis. Decision curve analysis (DCA) indicated that the prognostic factor + RS model had the best net benefit. Finally, nine tumor-infiltrating immune cells (TIICs) showed significant differences in abundance between high- and low-risk groups via CIBERSORT deconvolution algorithm. In conclusion, the immune-related eleven-RNA signature be could served as a potential prognostic biomarker for osteosarcoma metastasis.

## Introduction

Osteosarcoma is the most common malignant primary bone tumor in young adolescents and children^1^. Early metastasis and poor prognosis are the main features of osteosarcoma. Approximately 20% of patients exhibit metastasis at diagnosis ^2^. The current standard management of newly diagnosed osteosarcoma involves in surgery, neoadjuvant chemotherapy, and postoperative adjuvant chemotherapy, which has raised the 5-year survival of localized osteosarcoma to approximately 70%^3,4^. However, metastasis remains the main challenge in osteosarcoma therapy as a considerable cause of death in patients with osteosarcoma ^5^. The outlook of metastatic osteosarcoma seems to be grim as the 5-year survival drops to as little as 20% and has stagnated over the past few decades, underscoring the critical need for novel therapeutic strategies^6^. Metastasis is closely related to OS prognosis ^7^. Thus, the development of valuable prognostic biomarkers is imperative to improve the survival of osteosarcoma patients, especially in the case of metastatic osteosarcoma.

Immunotherapy is becoming an appealing application in osteosarcoma therapy^8^. The tumor microenvironment (TME) shows communication between tumors and immune cells and plays a strong role in the development of osteosarcoma ^9,10^. TIICs in the TME are reported to be relevant for the prognosis of osteosarcoma^11^. Thus, a comprehensive analysis of immune infiltration in osteosarcoma may contribute to identifying potential prognostic biomarkers for osteosarcoma. In this study, the immune infiltration of osteosarcoma metastasis was evaluated using several algorithms such as ESTIMATE and CIBERSORT. An immune-related RNA signature was identified as a prognostic marker through LASSO Cox regression analysis and used to build a risk score (RS) model to assess the prognosis of osteosarcoma metastasis. This study might enhance the knowledge of the clinical prognostic outcomes of osteosarcoma metastasis and provide new clues to the potential immune-related therapeutic targets for osteosarcoma metastasis.

## Methods

### Data collection and preprocessing

RNA-Seq expression data of GDC TCGA Sarcoma and phenotype data were downloaded from the UCSC Xena database, produced by the Illumina HiSeq 2000 RNA Sequencing platform. In total, 176 samples with expression data, metastatic diagnosis information, and survival data were obtained, which served as the training set for further analysis.

The GSE39055 dataset was downloaded from the NCBI Gene Expression Omnibus, and the platform used was the Illumina HumanHT-12 WG-DASL V4.0 R2 expression beadchip. This dataset was public on Jan 22, 2013^12^. A total of 36 samples with expression and survival data were obtained, which served as the validation set in the following analysis.

Using the annotation file of each platform, both sets were re-annotated to determine the expression levels of the lncRNAs and mRNAs.

### Immune infiltration analysis and grouping

ssGSEA was employed to perform immune infiltration analysis of osteosarcoma samples, which was implemented using the GSVA package in R3.6.1. The stromal score, immune score, estimate score, and tumor purity of the osteosarcoma samples were calculated using the ESTIMATE algorithm. According to the results, osteosarcoma samples were split into two immunity groups: Immunity_H and Immunity_L.

### Identification of differential expression RNAs (DERs)

According to metastatic diagnosis information, the samples were divided into metastatic and non-metastatic groups. DERs (including DElncRNAs and DEmRNAs) were screened between Immunity_H vs. Immunity_L groups as well as metastasis versus non-metastasis groups by the cutoff of |log_2_FC|> 0.263 and false discovery rate (FDR) < 0.05, using the limma package in R3.6.1. Two DER sets from different comparisons between groups were intersected to obtain the common DERs. Functional enrichment analysis including gene ontology (GO) biology process (BP) and kyoto encyclopedia of genes and genomes (KEGG) ^13^ was applied to the common DERs using DAVID. The threshold was set at *p* < 0.05.

### Identification of prognostic feature DERs and development of RS model for prognosis

In the training set, the survival package in R3.6.1 was utilized to perform univariate Cox regression analysis on the common DERs to identify significant prognostic DERs. Multivariate Cox regression analysis was performed to determine the significant independent prognostic DERs. The threshold was set at log-rank *p* < 0.05. LASSO Cox regression analysis was performed to select prognostic feature RNAs using the penalized package in R3.6.1. Based on the optimal prognostic RNA signature, an RS model was constructed using the following formula:

$${\text{RS }} = \, \sum \beta_{{{\text{RNAs}}}} \times {\text{Exp}}_{{{\text{RNAs}}}}$$

where β_RNAs_ represents the regression coefficient of the RNA in the LASSO model, while Exp_RNAs_ denotes the expression value of the RNA. Based on this formula, the RS value of each sample in both sets was computed. Samples were split into high- and low-risk groups with the median RS as the cutoff criterion. The two groups were then analyzed for survival using the Kaplan–Meier method, implemented using the survival package in R3.6.1. The RS model was validated using GSE39055.

### Identification of independent clinical prognostic factors

In the training set, univariate and multivariate Cox regression analyses were applied to screen for independent clinical prognostic factors using the survival package in R3.6.1. The threshold was a log-rank *p* < 0.05. The results were visualized using the forest plot package in R3.6.1. Decision curve analysis (DCA) was then applied to the single prognostic factor, RS, and prognostic factors + RS models, which were implemented using the rmda package in R3.6.1, to observe the net benefit of each model, thereby analyzing the effect of different factors on prognosis.

### Correlational analysis between prognostic feature RNAs and TIIC types

In the training set, the abundance of 22 TIIC types was assessed using the CIBERSORT deconvolution algorithm. A t-test was used to select TIIC types with significant differences in abundance between the high- and low-risk groups. The correlation between the expression of prognostic feature RNAs and TIIC types was measured using Pearson’s correlation coefficient, which was calculated using the cor function in R3.6.1.

## Results

### Immune infiltration assessment and grouping

**Figure 1:**
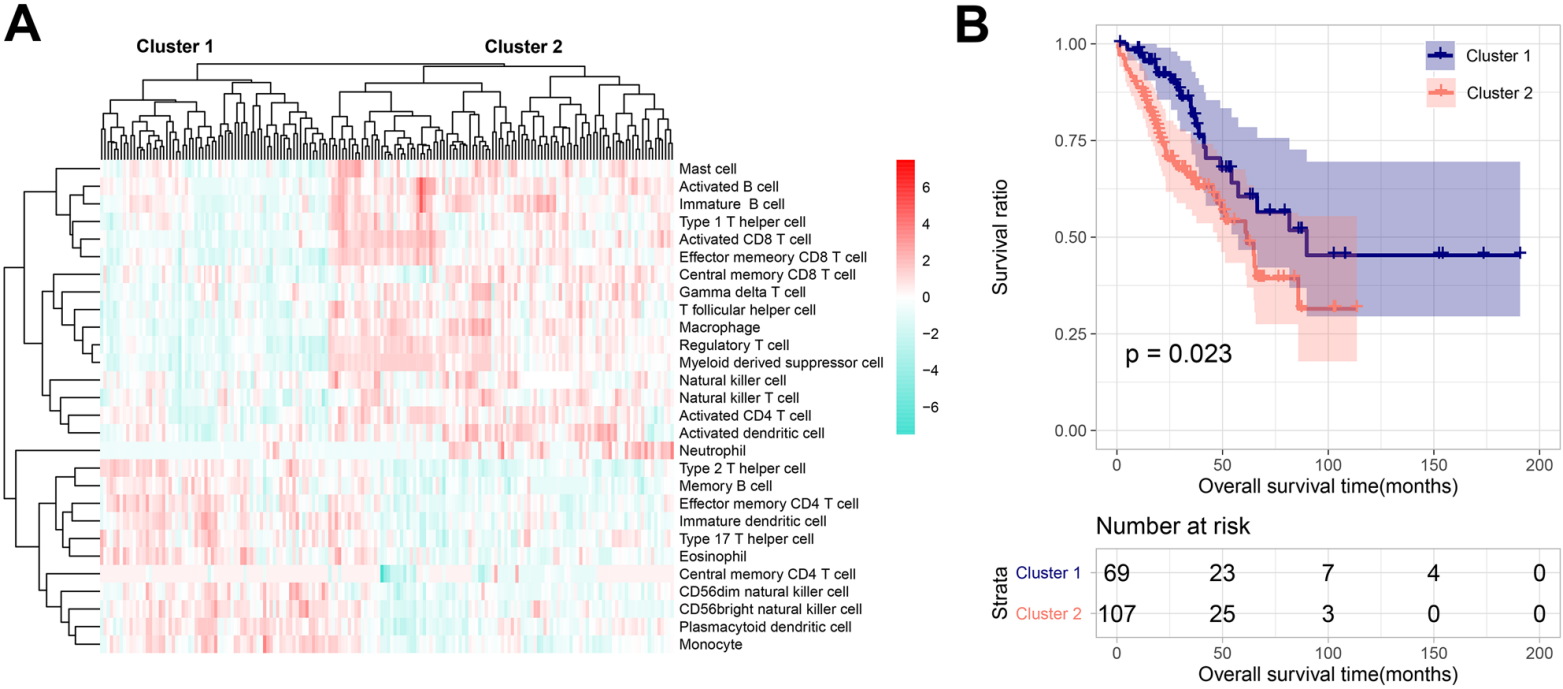
The hierarchical clustering results of immune infiltration of osteosarcoma samples based on ssGSEA (**A**) and Kaplan–Meier curves of survival of different clusters (**B**).

**Figure 2:**
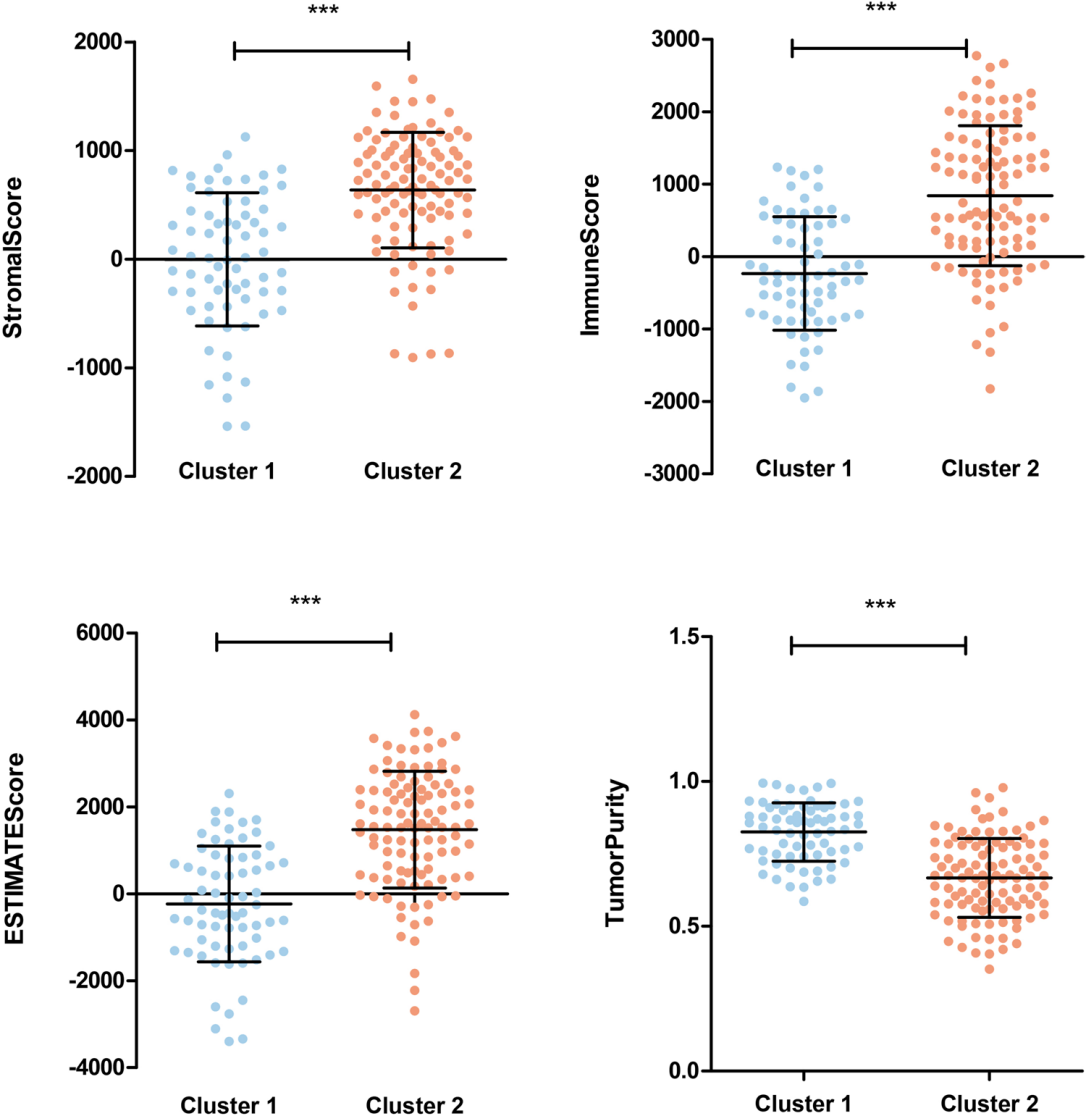
The distribution of stromal score, immune score, estimate score, and tumor purity of different clusters.

The flow diagram was shown in Fig. S1. After re-annotation, the expression data of 1398 lncRNAs and 14,631 mRNAs in the training set were obtained. Using ssGSEA, the samples were divided into two clusters (Fig. 1A). Cluster 1 included 69 samples, whereas Cluster 2 had 107 samples. Cluster 1 exhibited more favorable overall survival (Fig. 1B). As depicted in Fig. 2, the stromal, immune, and estimated scores were higher in cluster 2 than in cluster 1, while tumor purity was markedly lower. According to the assessment scores, Cluster 1 was defined as the Immunity_L group, whereas Cluster 2 was defined as the Immunity_H group.

### DERs and functional enrichment

A total of 542 DERs were obtained between the metastatic and non-metastatic groups (Fig. S2A). Meanwhile, 3624 DERs were obtained between the Immunity_H and Immunity_L groups Fig. S2B). A total of 268 common DERs, including 13 DElncRNAs and 255 DEmRNAs, were found between the two DER sets (Fig. S2C). Functional annotation showed that DEmRNAs were significantly enriched in 14 GO biological processes (Fig. S3A) and 12 KEGG pathways (Figure S3B), such as the immune response and B cell receptor signaling pathway.

### RS prognostic model

In the training set, 86 DERs were identified to be significantly related to prognosis via univariate Cox regression analysis, including seven DElncRNAs and 79 DEmRNAs. Through multivariate Cox regression analysis, 60 significant independent prognostic DERs were screened, consisting of 6 DElncRNAs and 54 DEmRNAs. Furthermore, 11 DERs were identified as prognostic feature RNAs (Fig. S4A,B), comprising two DElncRNAs (LINC00324 and LINC00672) and nine DEmRNAs (MAP3K8, SLC2A6, ABCB1, C1orf127, RIPK3, GGT1, RAB7B, BDKRB1, and APCDD1L).

Based on the optimal prognostic RNA signature, the RS model was built as follows:

$$\begin{aligned} {\text{RS}} = & \left( { - 0.0{377}0{3623}} \right) \, *{\text{ Exp}}_{{{\text{MAP3K8}}}} + \left( { - 0.0{266162}0{9}} \right)*{\text{Exp}}_{{{\text{SLC2A6}}}} \\ & + \left( { - 0.0{23773466}} \right)*{\text{Exp}}_{{{\text{ABCB1}}}} + \left( { - 0.0{13457687}} \right)*{\text{ Exp}}_{{{\text{C1orf127}}}} \\ & + \left( { - 0.0{12}0{65782}} \right)*{\text{Exp}}_{{{\text{LINC}}00{672}}} + \left( { - 0.00{9766}0{63}} \right)*{\text{Exp}}_{{{\text{LINC}}00{324}}} \\ & + \left( { - 0.00{6871635}} \right)*{\text{Exp}}_{{{\text{RIPK3}}}} + \, \left( { - 0.00{5448974}} \right)*{\text{Exp}}_{{{\text{GGT1}}}} \\ & + \left( { - 0.00{257}0{943}} \right)*{\text{Exp}}_{{{\text{RAB7B}}}} + \, \left( { - 0.00{17}0{8685}} \right)*{\text{Exp}}_{{{\text{BDKRB1}}}} \\ & + \left( {0.00{5993841}} \right)*{\text{Exp}}_{{{\text{APCDD1L}}}} \\ \end{aligned}$$

**Figure 3:**
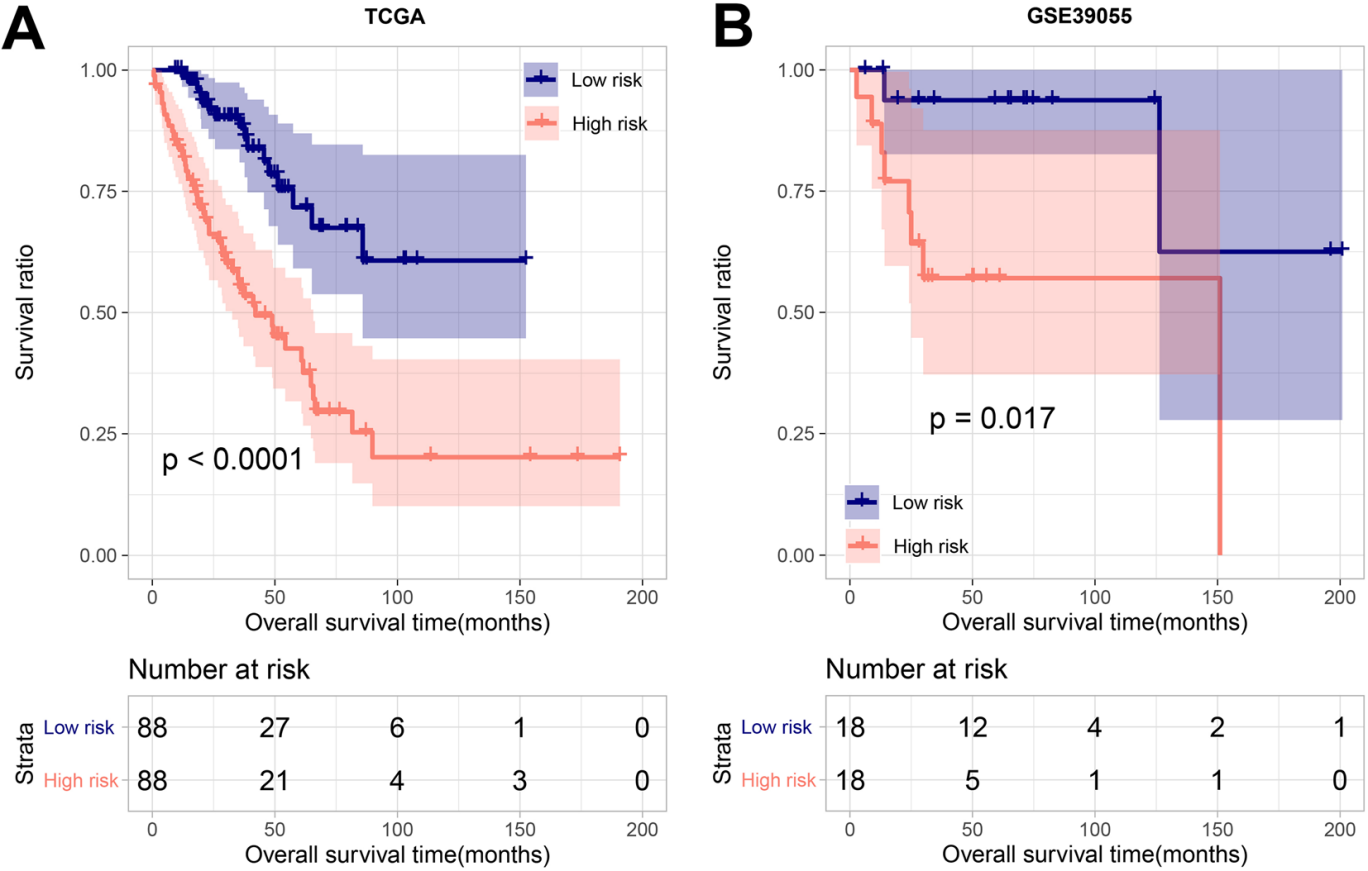
Kaplan–Meier curves of survival of high and low risk groups in the training set (**A**) and the validation set (**B**).

Based on the median RS, the samples were split into high- and low-risk groups in both training dataset (Fig. S5A) and validation dataset (Fig. S5B). The Kaplan–Meier curve revealed that the RS was markedly related to prognosis, and the high-risk group showed a shorter survival (Fig. 3A). Also, the above results were validated using GSE39055 dataset (Fig. S5B, Fig. 3B), suggesting that a high RS was notably relevant to poor prognosis.

### Independent clinical prognostic factors

**Figure 4:**
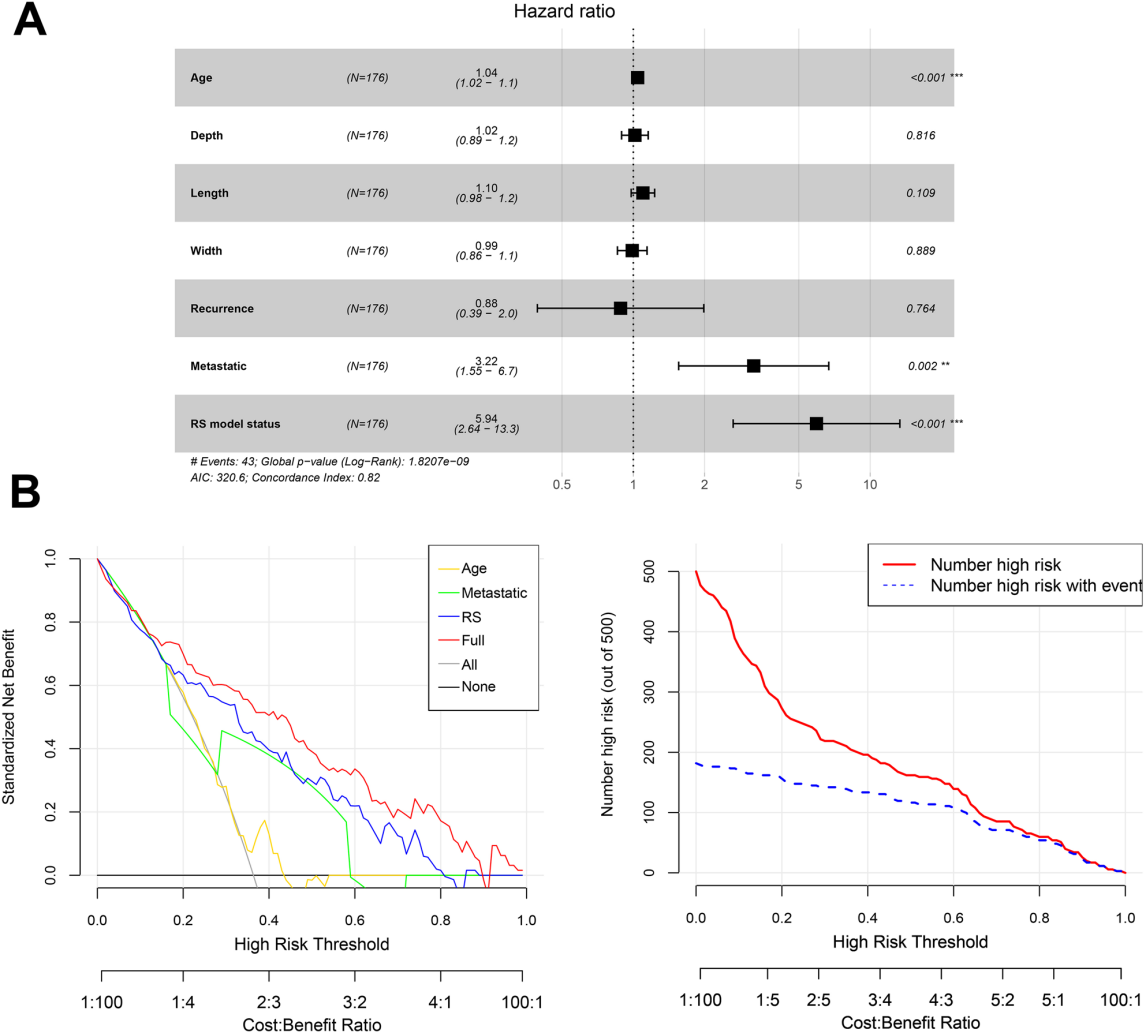
Identification of independent clinical prognostic factors (**A**) and decision curve analysis of different models (**B**).

Three independent clinical prognostic factors were identified: including age, tumor metastasis, and RS model status (Fig. 4A). DCA showed that, with respect to the net benefit, the prognostic factors + RS model was superior to other models in most ranges of the threshold probability, indicating that this prediction model had the best clinical utility (Fig. 4B).

### Correlational between prognostic feature RNAs and TIIC types

**Figure 5:**
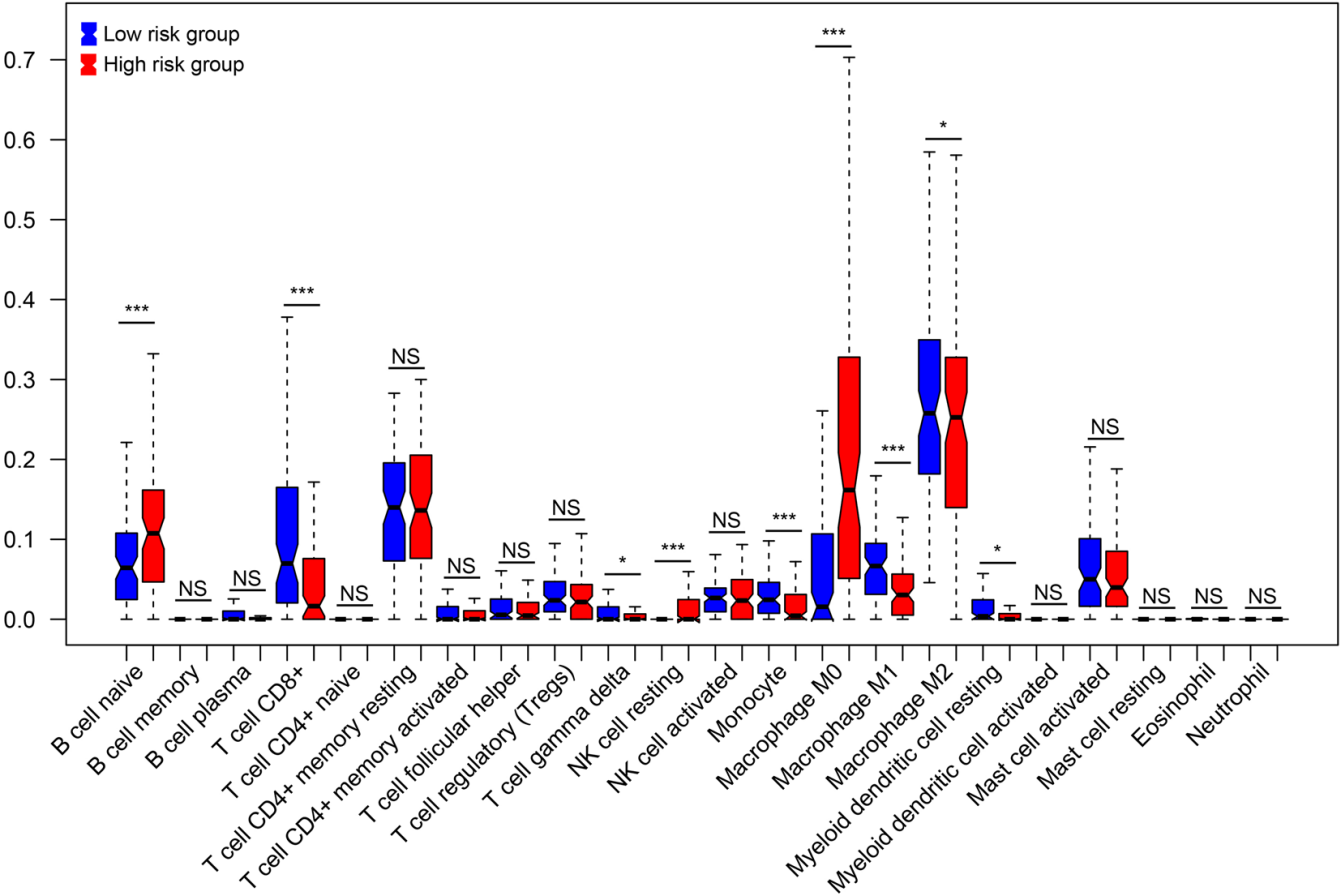
The abundances of 22 TIIC types between high and low risk groups.

**Figure 6:**
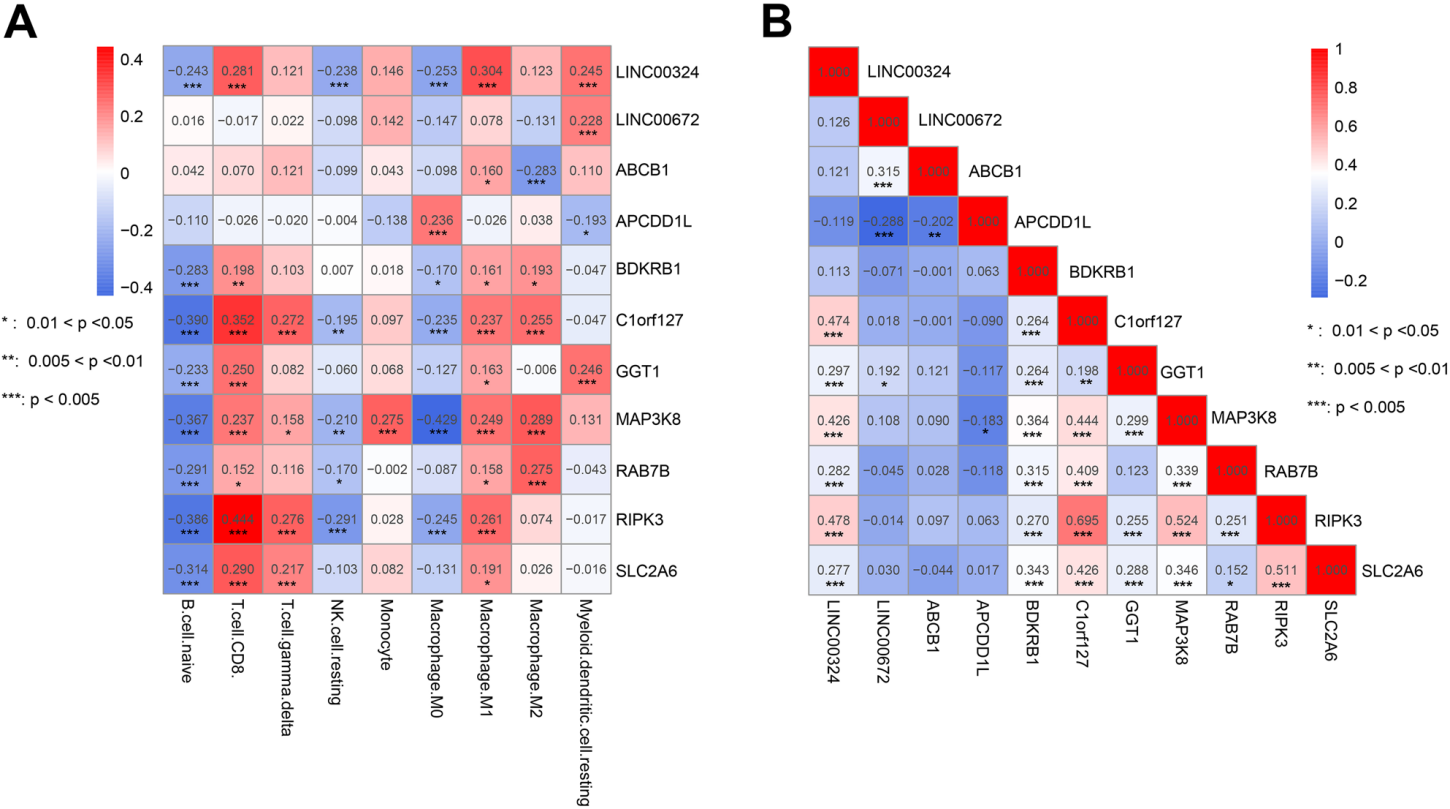
The correlation analysis. (**A**) The correlation matrix of 11 prognostic feature DERs and 9 TIIC types. (**B**) The correlation matrix of all the prognostic feature DERs.

Using the CIBERSORT deconvolution algorithm, 9 of 22 TIIC types exhibited significant differences in abundance between the high- and low-risk groups, including naïve B cell, CD 8 + T cells, γ δ T cells, monocytes, resting NK cells, M0 macrophages, M1 macrophages, M2 macrophages, and resting myeloid dendritic cells (Fig. 5). The correlation between 11 prognostic feature RNAs and 9 TIIC types was assessed by calculating the Pearson correlation coefficient (Fig. 6). For instance, MAP3K8 is associated with eight TIIC types, except for resting myeloid dendritic cells. MAP3K8 is the only gene related to monocytes. The expression of MAP3K8 was relevant to that of LINC00324, C1orf127, GGT1, BDKRB1, and APCDD1L.

## Discussion

Osteosarcoma is a high-grade malignant bone tumor with a low overall survival (OS) rate. As one of the key determinants of the lethality of osteosarcoma, metastasis usually leads to a poor prognosis, with only about 20% becoming long-term survivors compared to 70 that of patients with localized disease. Treatment modalities and clinical consequences for osteosarcoma have remained unchanged for decades, prompting the interest in novel strategies for osteosarcoma management and therapy. Immunotherapy has gained attention for osteosarcoma ^14,15^. The exploration of valuable immune-related prognostic biomarkers is beneficial for predicting high-risk patients and improving immunotherapy efficacy to improve the long-term survival outcomes of patients with osteosarcoma metastasis.

In the present study, 11 immune-related independent prognostic RNAs were used to generate an RS model to predict the prognosis of osteosarcoma samples. The AUC values of the RS model for assessing 1-, 3-, and 5-year survival were 0.958, 0.927, and 0.920 in the training set, indicating that the RNA signature exhibited a good capability for predicting survival in osteosarcoma samples, which was also verified in the validation set. Cases were divided into high- and low-risk groups based on the median RS. RS was significantly associated with prognosis. Patients in the high-risk group showed poor survival outcomes. More than 60% of the samples in the high-risk group died within 5 years, whereas in the low-risk group, about 25% died. Therefore, appropriate monitoring and intervention should be considered for high-risk groups, for instance, more continuous follow-up visits and active treatments.

The RS model status, tumor metastasis, and age were served as independent prognostic factors for osteosarcoma survival in the current study. It is well admitted that metastasis is one of the most disadvantages among known prognostic factors at diagnosis ^16^. The lung is the main site of osteosarcoma metastasis, accounting for approximately 80% of all metastatic cases. Osteosarcoma also metastasizes to bones and lymph nodes. As a multistep complex process that involves invasion, intravasation, dissemination, extravasation, and colonization, metastasis is difficult to control^17^. Despite the introduction of multimodal chemotherapy and surgery, the dismal long-term survival of osteosarcoma metastasis remains for decades ^18^. Bimodal age distribution is a characteristic of osteosarcoma, the first peak of which is documented in young children and adolescents, and the second peak is observed in geriatric patients^19^. The first peak is attributed to intense linear bone growth, whereas the second peak is associated with increased bone resorption by osteoclasts^18^. Recently, a meta-analysis demonstrated that older osteosarcoma patients fare worse than younger^20^. However, other studies have shown that age is not a significant independent prognostic factor, which blurs the prognostic role of age in osteosarcoma^21^. Some are optimistic that with better medical technology, age will cease to be a vital factor in prognosis^22^.

The immune infiltration landscape of the osteosarcoma samples was observed using the CIBERSORT algorithm, and nine TIICs exhibited significant differences in abundance between the high- and low-risk groups. Macrophages are reported to be the primary members of the immune environment in osteosarcoma^23^, which is in line with our results. Monocytes, as precursors of macrophages, can enter tumor tissues and polarize into M1 or M2 macrophages in the TME^24^. In the current study, the high-risk group that showed poor survival had increased levels of M0 macrophages and markedly decreased levels of monocytes and M1 and M2 macrophages, implying that phenotypic changes and polarization levels of macrophages may be associated with the osteosarcoma survival. Zhang et al.^25^ a similar view that the polarization of M0 to M1 or M2 macrophages is relevant to the ameliorative outcome of osteosarcoma. Moreover, M2 macrophages are related to osteosarcoma metastasis and poor patient prognosis, whereas a phenotype change from M2 to M1 macrophages causes the regression of lung metastasis of osteosarcoma ^26,27^. Some strategies targeting macrophages have displayed a bright future in osteosarcoma therapy, such as blocking the polarization of M1 to M2 macrophages or inhibiting M2 macrophages directly, activating macrophages, and enhancing non-macrophage recruitment^28^. Further basic experiments and clinical investigations focusing on immunotherapy are warranted to improve the survival of osteosarcoma patients.

Bioinformatics plays a crucial role in the discovery of new potential biomarkers for cancer by integrating omics data from various sources. Omics data, including genomics, transcriptomics, proteomics, and metabolomics, provide a comprehensive view of the molecular mechanisms underlying cancer development and progression.

Integration of omics data allows researchers to uncover complex interactions between different molecular layers, leading to a more comprehensive understanding of the underlying biological processes in cancer. In this study, the bioinformatics was performed on the omics data to identify the novel biomarkers for osteosarcoma. This holistic approach facilitates the identification of novel biomarkers that can be used for early detection, prognosis, and prediction of treatment response in cancer patients.

## Conclusion

In conclusion, an immune-related eleven-RNA signature-derived risk score model performed well in assessing the prognosis of osteosarcoma metastasis, and these immune-related RNAs may be promising prognostic biomarkers and targets for osteosarcoma metastasis.

### Supplementary Information

Supplementary Figure S1.

Supplementary Figure S2.

Supplementary Figure S3.

Supplementary Figure S4.

Supplementary Figure S5.

Supplementary Information 6.

## Data Availability

The datasets used and/or analyzed during the current study are available from the corresponding author upon reasonable request.
