# Supplementary figures and images for: An immune-related eleven-RNA signature-drived risk score model for prognosis of osteosarcoma metastasis
Source: Sci Rep. 2024 Jun 11;14:13401. doi: 10.1038/s41598-024-54292-6 (PMC11166963; doi:10.1038/s41598-024-54292-6)

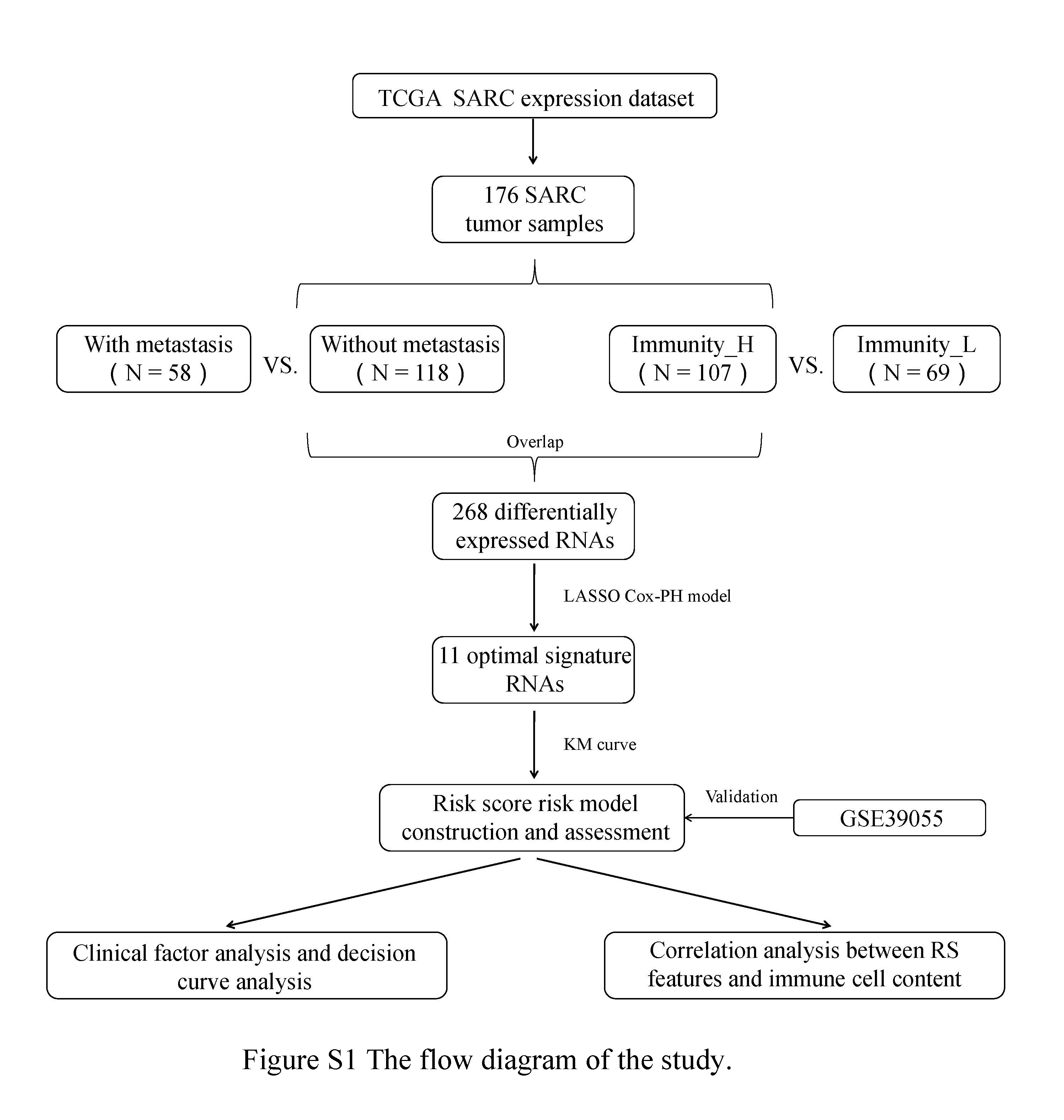

Supplement: Supplementary file 1 — Supplementary Figure S1. [file 41598_2024_54292_MOESM1_ESM.jpg]

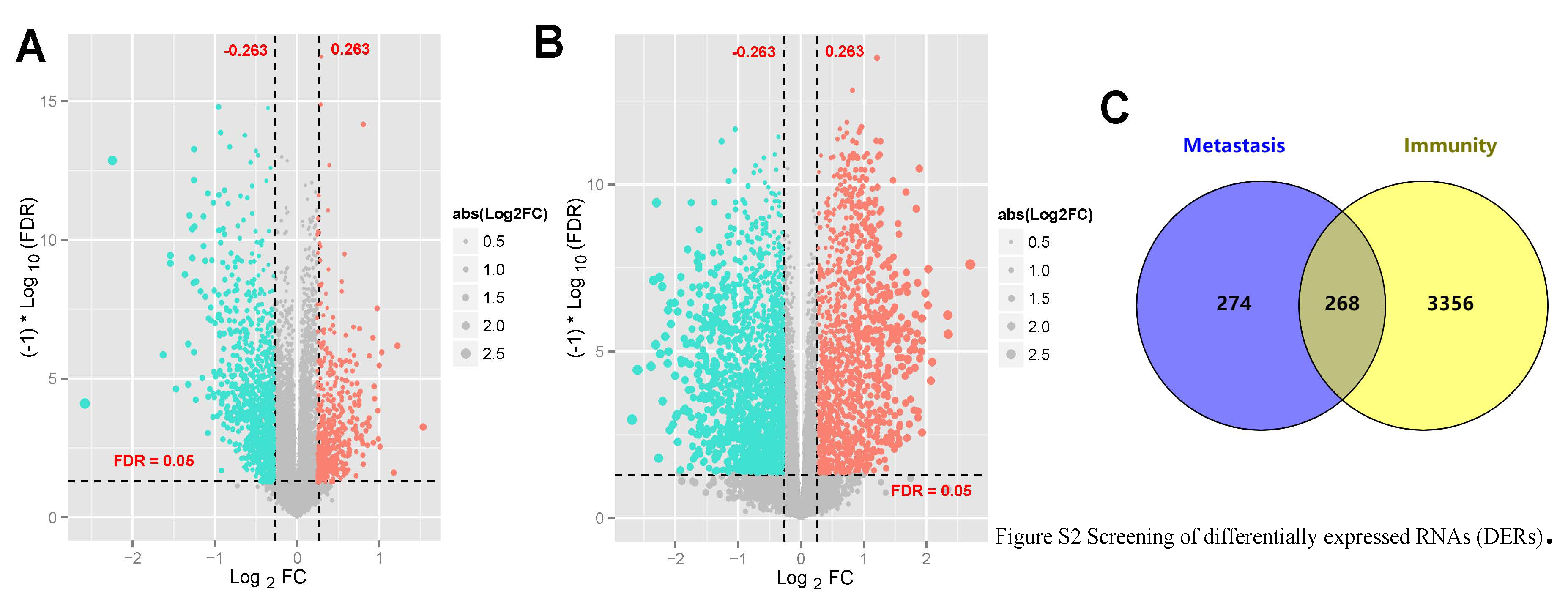

Supplement: Supplementary file 2 — Supplementary Figure S2. [file 41598_2024_54292_MOESM2_ESM.jpg]

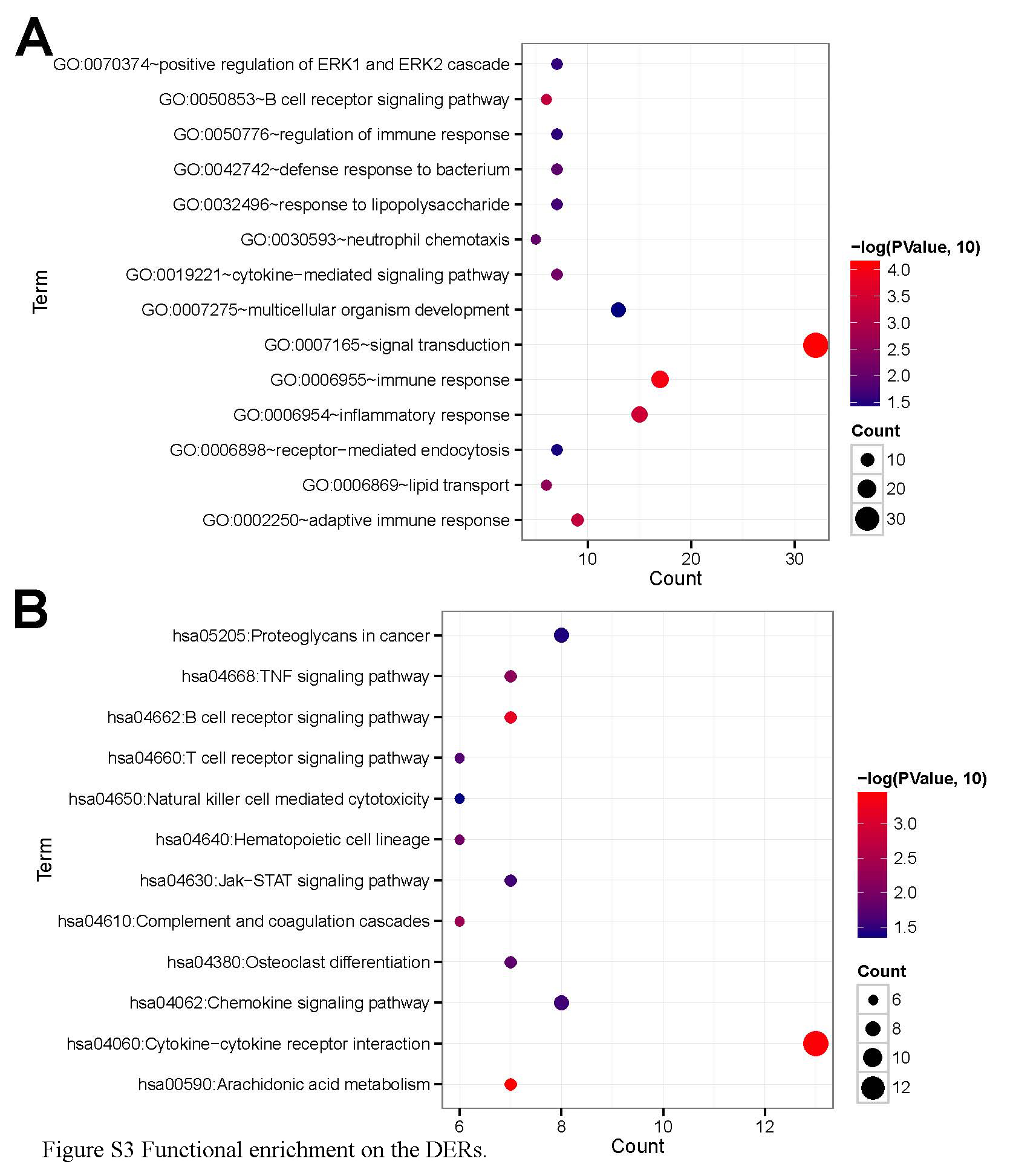

Supplement: Supplementary file 3 — Supplementary Figure S3. [file 41598_2024_54292_MOESM3_ESM.jpg]

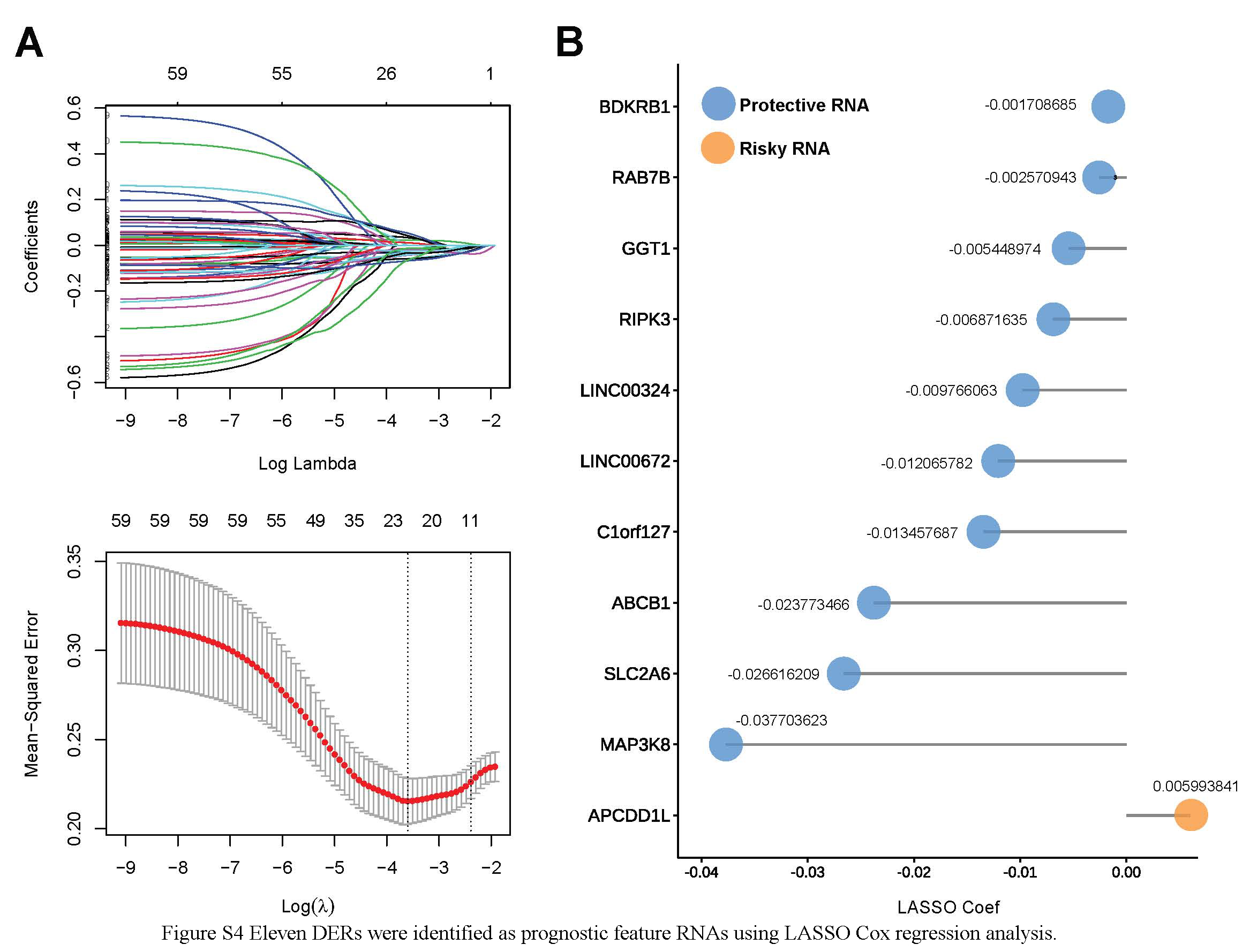

Supplement: Supplementary file 4 — Supplementary Figure S4. [file 41598_2024_54292_MOESM4_ESM.jpg]

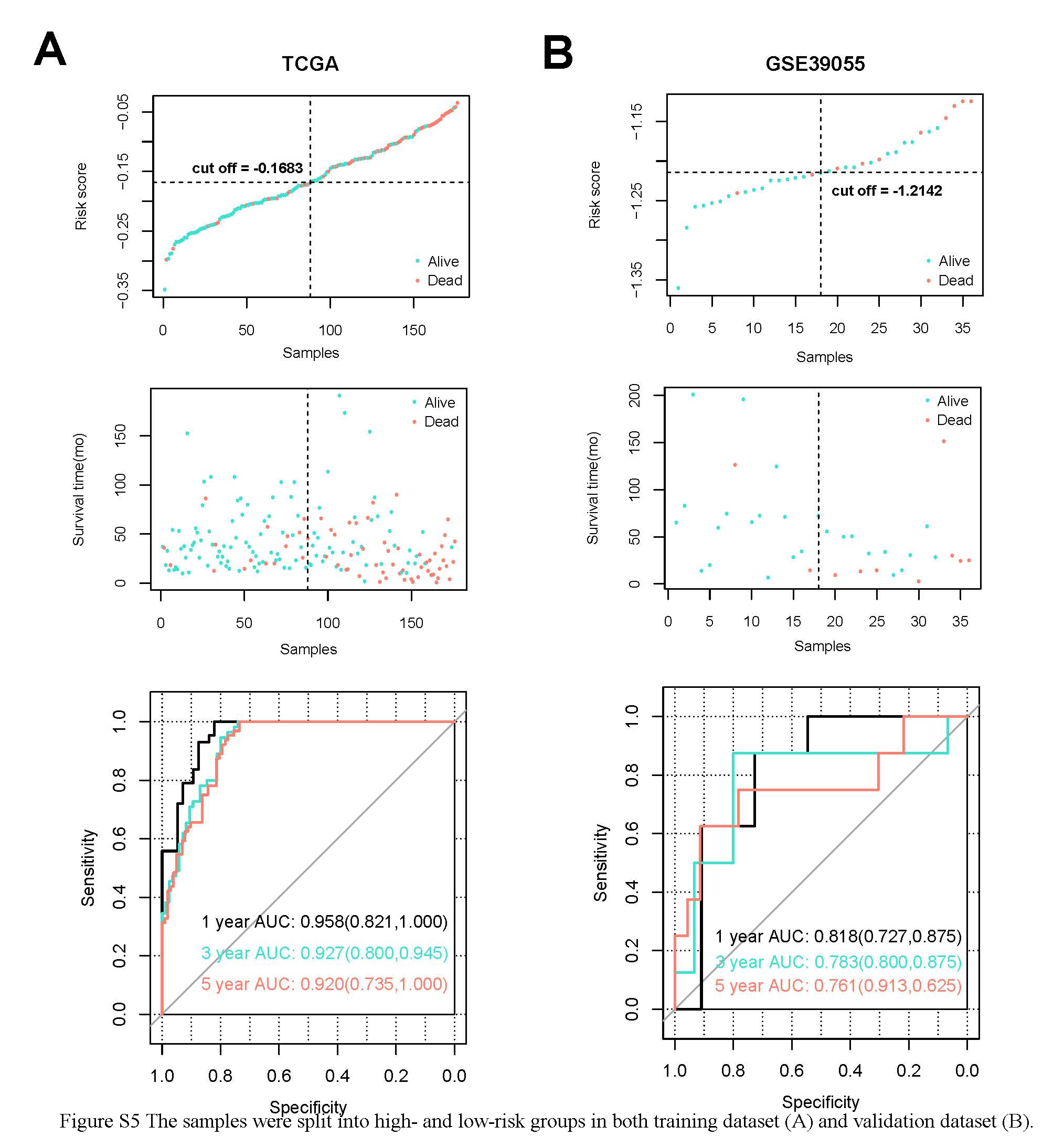

Supplement: Supplementary file 5 — Supplementary Figure S5. [file 41598_2024_54292_MOESM5_ESM.jpg]
